# Comparative genome analysis of the candidate functional starter culture strains *Lactobacillus fermentum* 222 and *Lactobacillus plantarum* 80 for controlled cocoa bean fermentation processes

**DOI:** 10.1186/s12864-015-1927-0

**Published:** 2015-10-12

**Authors:** Koen Illeghems, Luc De Vuyst, Stefan Weckx

**Affiliations:** Research Group of Industrial Microbiology and Food Biotechnology (IMDO), Faculty of Sciences and Bioengineering Sciences, Vrije Universiteit Brussel (VUB), Pleinlaan 2, B-1050 Brussels, Belgium

**Keywords:** *Lactobacillus fermentum*, *Lactobacillus plantarum*, Cocoa bean fermentation, 454 pyrosequencing, Functional starter cultures

## Abstract

**Background:**

*Lactobacillus fermentum* 222 and *Lactobacillus plantarum* 80, isolates from a spontaneous Ghanaian cocoa bean fermentation process, proved to be interesting functional starter culture strains for cocoa bean fermentations. *Lactobacillus fermentum* 222 is a thermotolerant strain, able to dominate the fermentation process, thereby converting citrate and producing mannitol. *Lactobacillus plantarum* 80 is an acid-tolerant and facultative heterofermentative strain that is competitive during cocoa bean fermentation processes. In this study, whole-genome sequencing and comparative genome analysis was used to investigate the mechanisms of these strains to dominate the cocoa bean fermentation process.

**Results:**

Through functional annotation and analysis of the high-coverage contigs obtained through 454 pyrosequencing, plantaricin production was predicted for *L. plantarum* 80. For *L. fermentum* 222, genes encoding a complete arginine deiminase pathway were attributed. Further, in-depth functional analysis revealed the capacities of these strains associated with carbohydrate and amino acid metabolism, such as the ability to use alternative external electron acceptors, the presence of an extended pyruvate metabolism, and the occurrence of several amino acid conversion pathways. A comparative genome sequence analysis using publicly available genome sequences of strains of the species *L. plantarum* and *L. fermentum* revealed unique features of both strains studied. Indeed, *L. fermentum* 222 possessed genes encoding additional citrate transporters and enzymes involved in amino acid conversions, whereas *L. plantarum* 80 is the only member of this species that harboured a gene cluster involved in uptake and consumption of fructose and/or sorbose.

**Conclusions:**

In-depth genome sequence analysis of the candidate functional starter culture strains *L. fermentum* 222 and *L. plantarum* 80 revealed their metabolic capacities, niche adaptations and functionalities that enable them to dominate the cocoa bean fermentation process. Further, these results offered insights into the cocoa bean fermentation ecosystem as a whole and will facilitate the selection of appropriate starter culture strains for controlled cocoa bean fermentation processes.

**Electronic supplementary material:**

The online version of this article (doi:10.1186/s12864-015-1927-0) contains supplementary material, which is available to authorized users.

## Background

Lactic acid bacteria (LAB) are Gram-positive bacteria that are key players in the majority of food fermentation ecosystems [1, 2]. The genus *Lactobacillus* represents the largest group among the LAB, encompassing more than 200 species [3, 4]. As this genus possesses a wide metabolic diversity, lactobacilli occur and have been used as functional starter cultures in a wide variety of fermented food products, including cheese, fermented plant-derived foods, fermented meats, wine and beer production, and sourdoughs [5]. Furthermore, lactobacilli play an important role during the cocoa bean fermentation process [6].

At the onset of the cocoa bean fermentation process, a wide LAB species diversity is often present [7–12]. However, only a restricted number of *Lactobacillus* species predominate this fermentation process, consisting mainly of (i) strictly heterofermentative *Lactobacillus fermentum* [7–22] and (ii) facultative heterofermentative *Lactobacillus plantarum* [7–12, 14–21, 23]. It has been proposed that *L. plantarum* is the dominant member at the onset of the cocoa bean fermentation process, while *L. fermentum* dominates in later stages [7, 8, 18, 21]. This is in accordance with the cocoa-specific functional roles that have been associated with these two species [24–26].

Homolactic fermentation of cocoa pulp-bean mass carbohydrates such as glucose and fructose, resulting from the hydrolysis of sucrose present in the cocoa pulp-bean mass by pulp or yeast invertase, results in lactic acid, while heterolactic fermentation gives lactic acid, acetic acid and/or ethanol, and carbon dioxide [6]. Fructose and citric acid, present in the cocoa pulp, are also used as alternative external electron acceptors by heterofermentative LAB, enhancing their competitiveness and resulting in the production of mannitol and succinate, or lactate and flavor-active compounds such as 2,3-butanediol or acetoin, respectively [24–26].

To better understand the exact role of cocoa-derived microorganisms, their whole-genome sequence can be investigated, as has been done for the cocoa-specific *Acetobacter pasteurianus* 386B strain [27]. Whole-genome sequencing of particular strains of the LAB species *L. fermentum* and *L. plantarum* originating from other sources (including fermented food ecosystems; Additional files 1 and 2) has been performed previously, thereby elucidating a plethora of specific functionalities and niche adaptations. For instance, genome sequence analysis of particular *L. fermentum* strains has revealed adaptations to specific host systems, such as the presence of adhesins [28] and probiotic properties [29–31]. Further, comparative genomics has enabled insights into the genomic features that are unique for *L. fermentum* strains [32]. Genome sequencing of particular *L. plantarum* strains elucidated niche adaptations [33, 34], probiotic properties [35–38], prophage occurrence [39–41], general metabolic properties [42–46], and bacteriocin production [47]. Therefore, it can be expected that whole-genome sequence analysis of the cocoa-derived *L. fermentum* 222 and *L. plantarum* 80 strains, isolates from a spontaneous Ghanaian cocoa bean fermentation process and proposed as interesting functional starter culture strains [7, 25, 26], will provide detailed insight into their genetic potential, metabolic capacities and functionalities as well as cocoa niche adaptations. Next to their central carbohydrate metabolism, their proteolytic system and amino acid conversion pathways have to be analysed, as these are important functionalities of LAB in various food fermentation ecosystems [48–50].

## Results and discussion

### 454 pyrosequencing, genome sequence assembly, and annotation

454 pyrosequencing of the genomic DNA of *L. fermentum* 222 and *L. plantarum* 80 yielded 571,369 reads with a median length of 435 base pairs (bp) and 621,544 reads with a median length of 416 bp, respectively (Table 1). The reads obtained from the genomic DNA of *L. fermentum* 222 were assembled into 73 contigs (>500 bp) with a 104-fold depth of coverage, whereas the reads obtained from the genomic DNA of *L. plantarum* 80 were assembled into 67 contigs (>500 bp) with a 65-fold depth of coverage. Furthermore, the genome sequences were estimated to be 2.2 million bp (Mb) with a guanine plus cytosine (G+C) content of 52.08 % for *L. fermentum* 222 and 3.6 Mb with a G+C content of 44.36 % for *L. plantarum* 80. For *L. fermentum* 222, these data were in accordance with other members of this species [29–31, 51]. However, the genome sequence size of *L. plantarum* 80 was larger compared with other members of this species, generally being around 3.2 Mb [39], indicating that the genome sequence of *L. plantarum* 80 encompassed one or more plasmids, which is common for this species [33, 37, 40, 41, 52]. Indeed, two genes encoding a plasmid replication initiator (*repA*) were found, located on a 49,183-bp (LP80_2408) and a 8,306-bp (LP80_3155) contig. Also, several contigs showed high sequence identity with the plasmid-specific PATRIC database, providing further evidence for the presence of plasmids. By automated annotation of the contigs obtained, 1,864 and 3,123 protein-encoding sequences (CDS) were found in the genome sequences of *L. fermentum* 222 and *L. plantarum* 80, respectively. Other relevant features deduced from the genome sequences are summarised in Table 1.

**Table 1:** General features of the reads, contigs, and draft genome sequences of *Lactobacillus fermentum* 222 and *L. plantarum* 80

Statistics	*L. fermentum* 222	*L. plantarum* 80
Reads		
Total bases	225,141,845	234,878,528
Number of reads	571,369	621,544
Average read length	394.65	378.34
Median read length	435	416
Assembly		
Number of contigs	73	67
N50 contigs	55,702	92,828
Depth of coverage	104	65
Average length	26,717	48,130
Longest contig	159,496	312,995
Draft genome sequences		
Estimated genome size (Mb)^a^	2.2	3.6
Total contig length (bp)	1,950,408	3,224,773
G+C content (%)	52.08	44.36
Number of CDS	1,864	3,123
Coding density (%)	83.00	83.00
Average gene length (bp)	869	857
Number of rRNA operons^b^	6	5
Number of tRNAs	54	67

^a^Estimation according to Newbler

^b^Estimation based on contig coverage

### Genome architecture and genetic potential

**Fig. 1:**
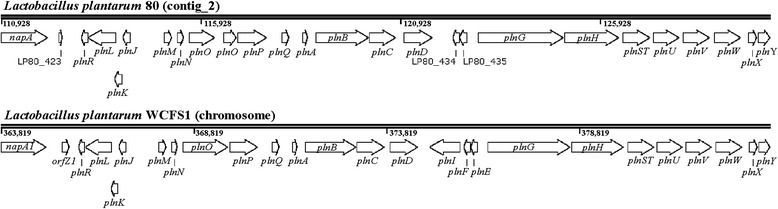
Genetic map of the *pln* loci of *Lactobacillus plantarum* 80 and *L. plantarum* WCFS1. For *L. plantarum* 80, a partial sequence of the 237,656-bp contig encoding the *pln* locus (LP80_422-LP80_443) is shown. For *L. plantarum* WCFS1, the chromosomal region containing the *pln* genes lp_0400-lp_0429 is shown

The genome sequences of *L. fermentum* 222 and *L. plantarum* 80 did not contain virulence factors, antibiotic resistance genes, or clustered regularly interspaced short palindromic repeat (CRISPR) sequences, confirming their applicability for food fermentation processes. One prophage-related genomic region was found in the genome sequence of *L. fermentum* 222 (LFER_1134-LFER_1187) and three such regions were found in the genome sequence of *L. plantarum* 80 (LP80_860-LP80_887, LP80_1614-LP80_1668, LP80_2307-LP80_2359). Although these regions were identified by PHAST analysis as ‘intact prophage’, these phage-associated regions might not be active or inducible, as has been reported before in LAB [51]. Further, genes involved in bacteriocin biosynthesis were not found in the genome sequence of *L. fermentum* 222. The genome sequence of *L. plantarum* 80 contained a plantaricin (*pln*) locus (LP80_422-LP80_443) on a 237,656-bp contig (Fig. 1), comprising all genes needed for the production of this bacteriocin [53]. BLASTn analysis of this contig using the plasmid-specific PATRIC database revealed that this plantaricin is chromosomally encoded in *L. plantarum* 80. This plantaricin locus was similar to that of *L. plantarum* WCFS1 [33], including a transport operon (*plnGHSTUVW*), all genes encoding a two-peptide bacteriocin JK (*plnJKLR*), a regulatory operon (*plnABCD*), a putative operon (*plnMNOP*), and genes encoding a putative toxin-antitoxin system (*plnXY*). *Lactobacillus plantarum* 80 did not contain the genes encoding the two-peptide bacteriocin EF (*plnEFI*), which were present in the *pln* locus of *L. plantarum* WCFS1. Instead, genes encoding putative proteins were found in the *pln* locus of *L. plantarum* 80 (Fig. 1; LP80_423, LP80_434-LP80_435). Production of plantaricin JK might enable *L. plantarum* 80 to outcompete other *L. plantarum* species present in the cocoa bean fermentation process, as this bacteriocin inhibits mainly the species *L. plantarum* [54, 55]. The genome sequence of *L. fermentum* 222 contained a 14-kb exopolysaccharide biosynthesis gene cluster (LFER_1022-LFER_1033), including a gene encoding a dextransucrase (LFER_1022), indicating that this strain is able to produce extracellular homopolysaccharides of the dextran type, possibly to protect itself.

Concerning adaptation to stress conditions, both genome sequences contained genes encoding an ATP synthase (*atpABCDEFGH*; LFER_671-LFER_678 for *L. fermentum* 222 and LP80_834-LP80_841 for *L. plantarum* 80), which enables regulation of the internal pH [56]. Further, the heat-shock operon *hrcA-grpE-dnaKJ* as well as several other heat-shock protein-encoding genes were found in both genome sequences (Table 2). Also, cold-shock protein-encoding genes as well as genes encoding Clp ATPases and proteases, which are involved in stress responses [57], were found in both genome sequences (Table 2). These cold-shock proteins might be part of the stress adaptation system of *L. plantarum* strains, as previously suggested for *L. plantarum* WCFS1 [33]. Finally, the genome sequence of *L. plantarum* 80 contained a *katA* gene (LP80_2743) encoding a heme-dependent catalase, which protects the cell from oxidative stress [58].

**Table 2:** Mechanisms of *Lactobacillus fermentum* 222 and *Lactobacillus plantarum* 80 involved in stress responses. The locus tags are depicted for each mechanism as well as the gene names when appropriate

Mechanism	*L. fermentum* 222	*L. plantarum* 80
Heat-shock operon *hrcA-grpE-dnaKJ*	LFER_1617-LFER_1620	LP80_2199-LP80_2202
Heat-shock proteins	LFER_1794	LP80_658; LP80_2456; LP80_2821
Cold-shock proteins	LFER_598	LP80_47; LP80_193; LP80_2125
Clp ATPases and proteases	*clpL* (LFER_111); *clpB* (LFER_572); *clpP* (LFER_1165, LFER_1522); *clpE* (LFER_1495); *clpX* (LFER_1563)	*clpB* (LP80_2653); *clpC* (LP80_170, LP80_2748); *clpE* (LP80_2599); *clpP* (LP80_1719, LP80_2338); *clpX* (LP80_923); *clpQY*; (LP80_1233-LP80_1234); *lon* (LP80_936)

## Metabolic pathway reconstruction

### Pathway analysis of *Lactobacillus fermentum* 222

#### Central carbohydrate metabolism

**Fig. 2:**
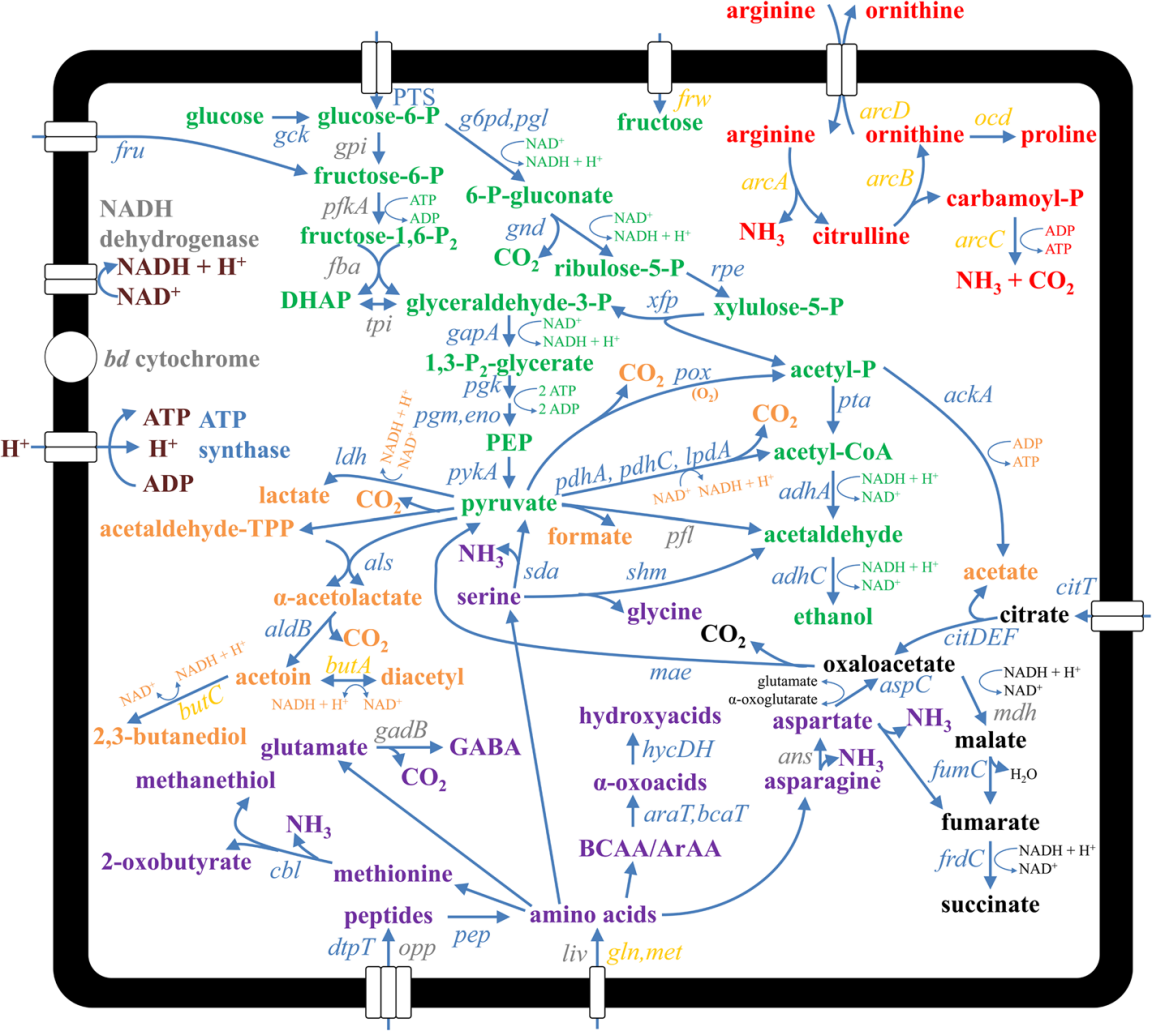
Metabolic pathways of *Lactobacillus fermentum* 222 and *Lactobacillus plantarum* 80. The names of the genes that were present in both genomes are depicted in blue, those that were present in *L. fermentum* 222 only are depicted in yellow, and those that were present in *L. plantarum* 80 only are depicted in grey. Metabolites involved in the homo- and heterolactic fermentation pathways are depicted in green, those involved in pyruvate metabolism are depicted in purple, those involved in citrate metabolism are depicted in black, those involved in the proteolytic system and amino acid conversion pathways are depicted in orange, those involved in the arginine deiminase pathway are depicted in red, and those involved in the respiratory machinery are depicted in dark red. Enzymes and locus tags that were associated with the gene names are listed in Additional file 3. *DHAP* Dihydroxyacetone phosphate, *PEP* Phosphoenolpyruvate, *BCAA*, Branched-chain amino acid, *ArAA* Aromatic amino acid, *GABA* γ-aminobutyric acid, *TPP* Thiamine pyrophosphate

All genes encoding the enzymes involved in the heterolactic fermentation pathway, responsible for the production of lactic acid, acetic acid and/or ethanol, were retrieved in the genome sequence of *L. fermentum* 222 (Fig. 2; Additional file 3). Further, different carbohydrate transport mechanisms were found, such as phosphoenolpyruvate (PEP)-dependent sugar phosphotransferase systems (PTS) and permeases. Pyruvate, originating from the heterolactic fermentation or the citrate metabolism (see below), may be used by *L. fermentum* 222 for NAD^+^ regeneration by production of diacetyl and/or acetoin, as the genes encoding α-acetolactate synthase (*als*), α-acetolactate decarboxylase (*aldB*), 2,3-butanediol dehydrogenase (*butC*), and diacetyl reductase (*butA*) were all found in the genome sequence (Fig. 2; Additional file 3). Alternatively, pyruvate may be converted into acetyl-CoA by the pyruvate dehydrogenase complex, with acetate or ethanol as the final products. Finally, a gene encoding pyruvate oxidase (*pox*) was found, enabling this strain to convert pyruvate into carbon dioxide and acetyl phosphate when oxygen is available. This requires reactive oxygen species tolerance, for which a gene encoding a thiol peroxidase (*tpx*; LFER_707) was found.

**Fig. 3:**
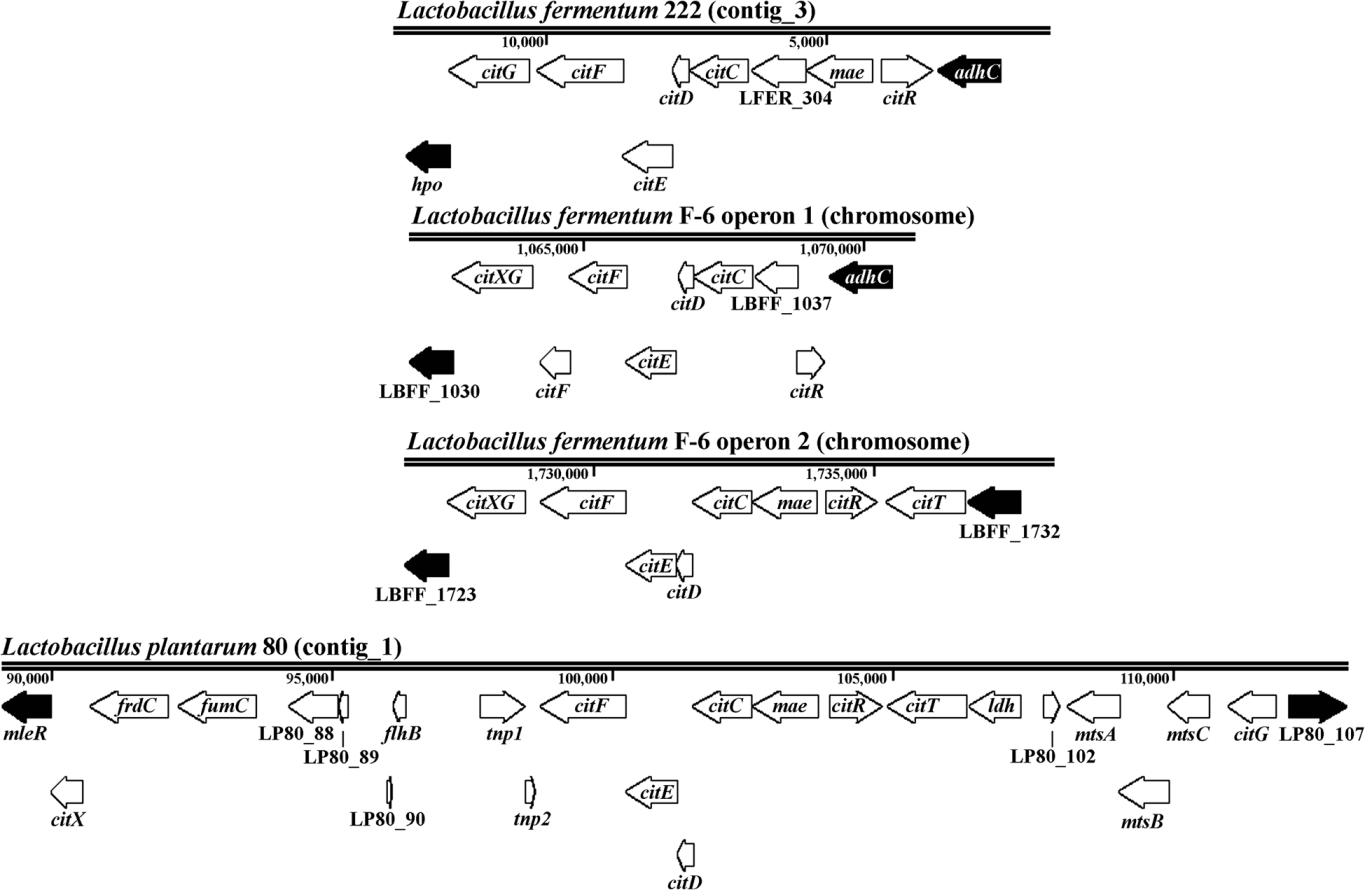
Organisation of the citrate operons in *Lactobacillus fermentum* 222, *L. fermentum* F-6, and *L. plantarum* 80. The appropriate genomic regions encoding the proteins involved in citrate uptake and conversion are shown for each genome sequence. For *L. fermentum* F-6, both citrate operons are depicted. Flanking genes outside the citrate operon are shown in black

A citrate operon, involved in the conversion of citric acid [26], was found in *L. fermentum* 222 (LFER_302-309; Figs. 2 and 3; Additional file 3), consisting of genes coding for a citrate lyase and its regulator (*citRDEF*), an oxaloacetate decarboxylating malate dehydrogenase (*mae*), a putative citrate transporter (LFER_304), and an accessory protein (citG*). In comparison with the two citrate operons that are encoded in the genome sequence of *L. fermentum* F-6, the only *L. fermentum* strain for which the whole-genome sequence is available harbouring citrate operons, the citrate operon of *L. fermentum* 222 showed differences with the ones of *L. fermentum* F-6. Indeed, the first citrate operon of *L. fermentum* F-6 seemed to be incomplete, as the *mae* gene was missing and the second operon had an atypical citrate transporter (Fig. 3) that was also found in *L. fermentum* 3872 [28], which is unlikely to be specific for citrate based on the sequence annotation of this gene. As such, the gene encoding this transporter was neither homologous to the one present in the citrate operon of *L. fermentum* 222 nor to the one in the other citrate operon of *L. fermentum* F-6. Besides the citrate transporter present in the citrate operon, two additional genes encoding a putative citrate transporter (LFER_11 and LFER_1857) were found in the genome sequence of *L. fermentum* 222. Yet, not all genes encoding the enzymes to convert oxaloacetate (produced by citrate lyase) into succinate were found, because only genes encoding fumarase (*fumC*) and fumarate reductase (*frdC*) were present, while a gene encoding malate dehydrogenase was missing.

Pathways that enable the use of oxygen or glycerol as alternative external electron acceptors were not found in the genome sequence of *L. fermentum* 222. Although this strain is able to use fructose as an alternative external electron acceptor [26] and the genome sequence contained a gene coding for a fructose permease (*frw*), a gene encoding a mannitol dehydrogenase could not be found. However, a fructose-specific PEP-dependent PTS (*fru*) was present, which is associated with mannitol production from fructose in homofermentative LAB [59]. Based on the genome sequence annotation, no further evidence was found that this transporter was involved in mannitol production.

### Proteolytic system and amino acid conversion pathways

The genome sequence of *L. fermentum* 222 harboured genes encoding a peptide transporter (*dtpT*) and several amino acid ATP-binding cassette (ABC) transporters specific for glutamine (*gln*), methionine (*met*), and several other amino acids that could not be further specified based on the genome sequence annotation (Fig. 2; Additional file 3). Further, a plethora of genes encoding peptidases were retrieved, such as *pepF* and *pepO* encoding endopeptidases, *pepI*, *pepP*, *pepQ*, and *pepX* encoding proline-specific peptidases, *pepD* and *pepV* encoding dipeptidases, *pepT* encoding a tripeptidase, and *pepC*, *pepN*, and *pepM* encoding aminopeptidases (Additional file 3). The acquisition of a large variety of peptidases by *Lactobacillales* is related to their adaptation to nutritionally rich environments [60], which might also be the case for members of the cocoa bean fermentation process, as cocoa pulp consists of 0.5–0.7 % proteins [6].

Aspartate transaminase-encoding genes were found (*aspC*), enabling *L. fermentum* 222 to produce oxaloacetate from aspartate. This oxaloacetate might contribute to the pyruvate metabolism through the pathway associated with citrate metabolism (see above). Indeed, it has been shown for other *Lactobacillus* species that diacetyl and acetoin can be produced through aspartate catabolism [61]. Genes encoding aspartase (*aspA*), fumarase (*fum*), and succinate dehydrogenase (*sdh*) were found as well, offering an alternative pathway for aspartate catabolism (Fig. 2; Additional file 3). This pathway may contribute to NAD^+^ regeneration, and, therefore, offer a more efficient degradation of carbohydrates [62].

Genes encoding the transaminases *araT* and *bcaT*, which catalyse the hydrolysis of branched-chain amino acids as well as aromatic amino acids, were retrieved (Fig. 2; Additional file 3). The concomitant α-oxoacids produced might be further reduced to hydroxyacids by the 2-hydroxyacid dehydrogenases (*hycDH*) present, thereby contributing to NAD^+^ regeneration and thus allowing more efficient energy production through heterofermentation, as hypothesised before [48]. Genes associated with the conversion of α-oxoacids by oxidative decarboxylation into carboxylic acids or by decarboxylation into aldehydes were not found. Neither were enzymes involved in decarboxylation of histidine and aromatic amino acids found, the latter indicating that *L. fermentum* 222 is unable of biogenic amine production. Further, evidence was found for the presence of other amino acid conversion pathways, such as the occurrence of genes encoding cystathionine β-lyase (*cbl*) that is involved in methionine catabolism, serine deaminase (*sda*) that enables the production of pyruvate and ammonia from serine, and serine hydroxymethyltransferase (*shm*) that is involved in the production of the flavour compound acetaldehyde (Fig. 2; Additional file 3).

The genome sequence of *L. fermentum* 222 contained an operon encoding the arginine deiminase (ADI) pathway, consisting of genes encoding arginine deiminase (*arcA*), ornithine transcarbamoyltransferase (*arcB*), carbamate kinase (*arcC*), an arginine/ornithine antiporter (*arcD*), and a transaminase (*arcT*; Fig. 2; Additional file 3). This pathway might provide *L. fermentum* 222 with additional energy, provided through amino acid conversions, as well as protection against acid stress conditions [63, 64] and has been shown to be of importance for competitiveness in food fermentation processes [64–66].

Further, a gene encoding ornithine cyclodeaminase (*ocd*) was present, enabling direct biosynthesis of proline from ornithine, thereby producing ammonia (Fig. 2; Additional file 3). The production of additional ammonia may further protect against acid stress, whereas proline can act as an osmotic protectant [67].

### Pathway analysis of *Lactobacillus plantarum* 80

#### Central carbohydrate metabolism

The genome sequence of *L. plantarum* 80 contained all genes encoding the enzymes involved in the homo- and heterolactic fermentation pathways (Fig. 2; Additional file 3) as well as various genes encoding carbohydrate uptake systems, enabling this strain to switch between both metabolic pathways, depending on the energy source available. Metabolic pathway analysis revealed that pyruvate can be converted into acetyl-CoA by a pyruvate dehydrogenase complex (*pdhAC, lpdA*) or by pyruvate-formate lyase (*pfl*), the latter yielding formate (Fig. 2; Additional file 3). Further, a gene encoding pyruvate oxidase (*pox*) was present, enabling *L. plantarum* 80 to convert pyruvate into carbon dioxide and acetyl phosphate under oxic conditions. Genes encoding α-acetolactate synthase (*als*) and α-acetolactate decarboxylase (*aldB*) were found, but diacetyl reductase- or 2,3-butanediol dehydrogenase-encoding genes were absent, indicating that acetoin would be the only flavour compound that can be formed out of pyruvate by *L. plantarum* 80, although 2,3-butanediol nor acetoin were found during monoculture fermentations with *L. plantarum* 80 in cocoa pulp simulation medium for LAB [26].

Genes associated with uptake and conversion mechanisms of citrate, such as a gene encoding citrate lyase (*citDEF*), oxaloacetate decarboxylating malate dehydrogenase (*mae*), a citrate transporter (*citT*), and accessory proteins (*citGX*), were all retrieved in the genome sequence of *L. plantarum* 80 (Fig. 2; Additional file 3). However, these were not clustered together (Fig. 3), as genes encoding transposases (LP80_92, LP80_93), an ABC transporter (*mtsABC*), a fumarase (*fumC*) and a fumarate reductase (*frdC*) were found between them. Further, a gene encoding a malate dehydrogenase (*mdh*) was found, which might enable *L. plantarum* 80 to use citrate as an alternative external electron acceptor by converting citrate into succinate by the reductive tricarboxylic acid cycle, next to its ability to convert citrate into pyruvate, which has been shown experimentally [27]. *Lactobacillus plantarum* 80 might be able to use oxygen as an alternative external electron acceptor, as a NADH-dependent oxidase-encoding gene (*nox*) was found. Genes associated with the ability to use glycerol or fructose as alternative external electron acceptors were absent.

The genome sequence of *L. plantarum* 80 harboured genes encoding a respiration machinery, which is an energetically favourable metabolic pathway that leads to less oxidative and acid stress in LAB [68]. Indeed, genes encoding a NADH-dependent dehydrogenase, a *bd*-type cytochrome (*cydABCD*), an ATP synthase (see above), and a heme-dependent catalase (*katA;* see above) were found (Fig. 2; Additional file 3). Although several *L. plantarum* strains possess a similar electron transport chain [69, 70], there are no experimental data indicating that *L. plantarum* 80 could perform aerobic respiration when provided with exogenous sources of heme and menaquinones.

#### Proteolytic system and amino acid conversion pathways

Genes encoding extracellular proteases were not found in the genome sequence of *L. plantarum* 80, which seems to be a general feature for *L. plantarum* strains [33]. Genes encoding a DtpT proton motive force-dependent peptide transporter and an Opp transporter (*oppABCDF*) were found as well as peptidase-encoding genes, including the genes *pepD*, *pepE*, and *pepV* encoding dipeptidases, the genes *pepN* and *pepC* encoding aminopeptidases, the genes *pepX*, *pepR*, and *pepP* encoding proline-specific peptidases, and the genes *pepF*, *pepQ*, *pepO*, and *pepR* encoding endopeptidases (Fig. 2; Additional file 3). This elaborate peptide uptake and degradation machinery might be an important mechanism for *L. plantarum* 80 to thrive in the protein-containing fermenting cocoa pulp-bean mass. Further, several genes encoding various amino acid transporters were present, such as *livABCDE*, enabling *L. plantarum* 80 to take up a variety of amino acids from the environment.

The genome sequence of *L. plantarum* 80 contained genes encoding an asparaginase (*ans*), an aspartate transaminase (*aspC*), and an aspartase (*aspA*; Fig. 2; Additional file 3). This pathway enables production of oxaloacetate and/or fumarate, which can subsequently be used for energy production and/or NAD^+^ regeneration by the enzymes encoded in the citrate operon (Fig. 3). Although a gene encoding glutamate decarboxylase was found (*gadB*), this pathway was unlikely to be operational, as the genes encoding the glutamate-γ-aminobutyric acid antiporter (*gadC*) and the transcriptional regulator (*gadR*) were not found, which are required for glutamate decarboxylation activity in other LAB [71].

A gene encoding the α-oxoglutarate-dependent aminotransferase BcaT was found in the genome sequence of *L. plantarum* 80, enabling the production of α-oxoacids from branched-chain amino acids. Further degradation of the α-oxoacids into hydroxyacids by this strain is feasible, as the genome sequence harboured a gene encoding a 2-hydroxyacid dehydrogenase (Fig. 2; Additional file 3). Enzymes involved in the conversion of α-oxoacids by oxidative decarboxylation into carboxylic acids or by decarboxylation into aldehydes were not found in the genome sequence. Further, the aromatic aminotransferase AraT was present in the genome sequence, which enables *L. plantarum* 80 to transaminate aromatic amino acids. In addition, aromatic amino acid decarboxylation enzymes were not found, indicating that *L. plantarum* 80 is not able to produce biogenic amines.

Genes encoding cystathionine β-lyase (*metC*) and cysteine kinase (*cysK*) were found in the same locus, which indicated that the methionine and cysteine catabolic pathways of *L. plantarum* 80 are linked, as has been shown previously for other LAB [72, 73]. Further, genes associated with the catabolism of serine and threonine, *i.e.*, a serine deaminase (*sda*) and a serine hydroxymethyltransferase (*shm*), were retrieved in the genome sequence of *L. plantarum* 80 (Fig. 2; Additional file 3). Genes involved in histidine decarboxylation or the ADI pathway were absent.

## Comparative genome sequence analysis

### Comparative genomics of *Lactobacillus fermentum* 222

The genome sequence of *L. fermentum* 222 harboured 182 strain-specific genes, *i.e.*, genes without a homolog in other known *L. fermentum* genome sequences (Additional file 1). Most of these genes encoded hypothetical proteins or were disrupted, although some had a predicted function. The strain-specific genes included two putative citrate transporters (LFER_11 and LFER_1857, discussed above) and a gene cluster (LFER_810-LFER_816) containing a diacetyl reductase-encoding gene (LFER_814; Fig. 2; Additional file 3). The complete *cit* gene cluster that was not interrupted by other genes or transposable elements (discussed above) and the occurrence of two unique additional (putative) citrate transporters might be the result of specific adaptations that enable this strain to consume citrate in an efficient way during the cocoa bean fermentation process [25, 74]. Further, a gene cluster involved in amino acid uptake and catabolism, consisting of genes coding for an amino acid transporter (LFER_135), an aspartase (LFER_136, discussed above), an amino acid permease (LFER_137), and an aminotransferase (LFER_138, discussed above) was unique for *L. fermentum* 222, providing evidence that amino acid conversion pathways might be an important feature for functional starter culture strains that have to improve the cocoa bean fermentation process.

**Fig. 4:**
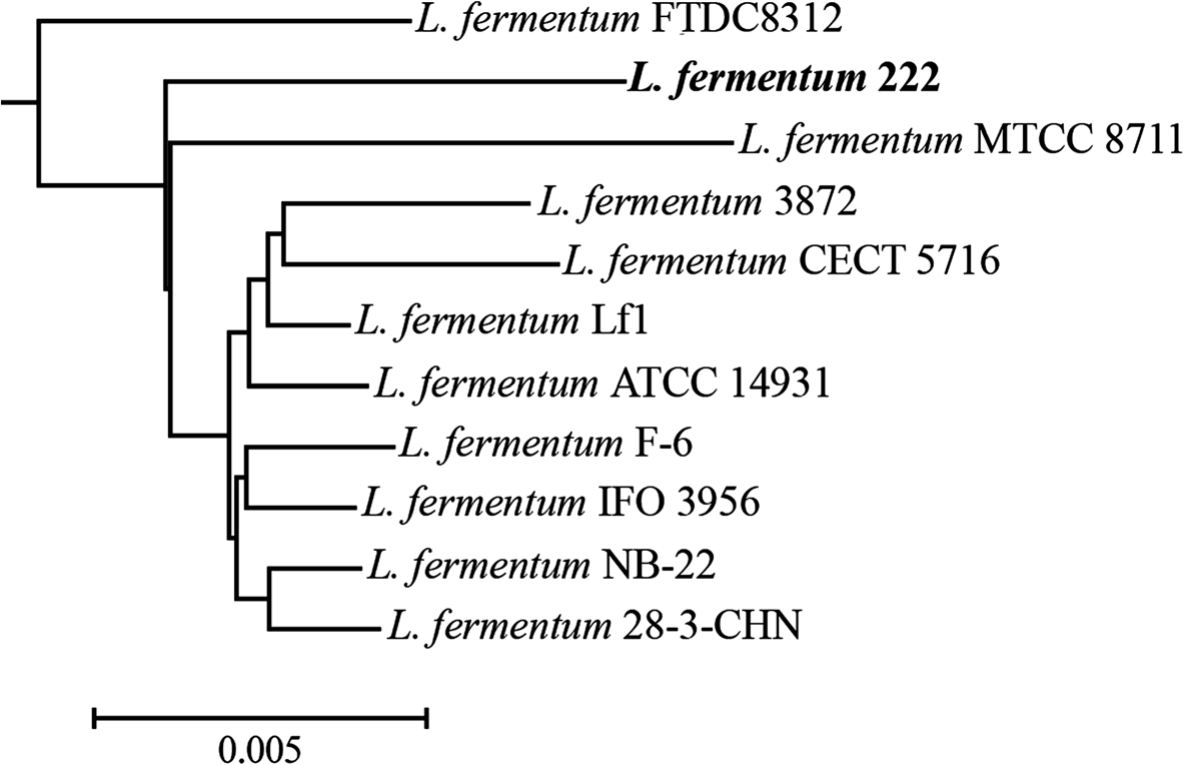
Phylogenetic tree based on all core gene sequences of *Lactobacillus fermentum* available. Multiple sequence alignments of concatenated core gene sequences were calculated using the EDGAR framework, including plasmid sequences. The genome sequence of *L. plantarum* 80 was used as an outgroup

The core genome of the species *L. fermentum*, based on eleven complete and draft genome sequences available (Additional file 1), consisted of 400 genes. The pan-genome of the species *L. fermentum* was found to encompass 4,475 genes and seemed to be open, *i.e.*, the addition of more strains in the comparative analysis would result in a larger pan-genome [75]. The latter result was in agreement with the fact that this species has been found in various environments, including mammalian body sites [28, 31, 76] and food (fermentation) environments [2, 30, 31]. A phylogenetic tree based on the core gene sequences revealed that *L. fermentum* 222 was not grouped together with any other *L. fermentum* strain (Fig. 4), which might indicate its specific adaptation to the cocoa bean fermentation ecosystem.

### Comparative genomics of *Lactobacillus plantarum*

A total of 223 strain-specific genes were found in the genome sequence of *L. plantarum* 80 compared to the currently available *L. plantarum* genome sequences (Additional file 2). Among these strain-specific genes was a gene cluster (LP80_2381-LP80_2387) consisting of the PTS-encoding *sorABCDE* genes, a dehydrogenase-encoding gene (*sorF*), and a gene encoding the transcriptional regulator (*sorR*). In *Lactobacillus casei*, this gene cluster is involved in transport of sorbose and subsequent production of fructose 6-phosphate, which can be channeled into the glycolytic pathway [77]. This mechanism can also be used to transport fructose, as shown for *L. casei* and *Klebsiella pneumoniae* [78]. These genes might thus offer *L. plantarum* 80 unique features to be able to dominate the fructose-containing cocoa pulp-bean mass during cocoa bean fermentation. Furthermore, a unique cluster of four genes was found, consisting of an oxidoreductase (LP80_2995), a ribose 5-phosphate isomerase (LP80_2996), a fructose-specific PTS (LP80_2997), and a transcriptional regulator (LP80_2998). These traits underline the fructophilic character of *L. plantarum* 80 during cocoa bean fermentation processes [26].

**Fig. 5:**
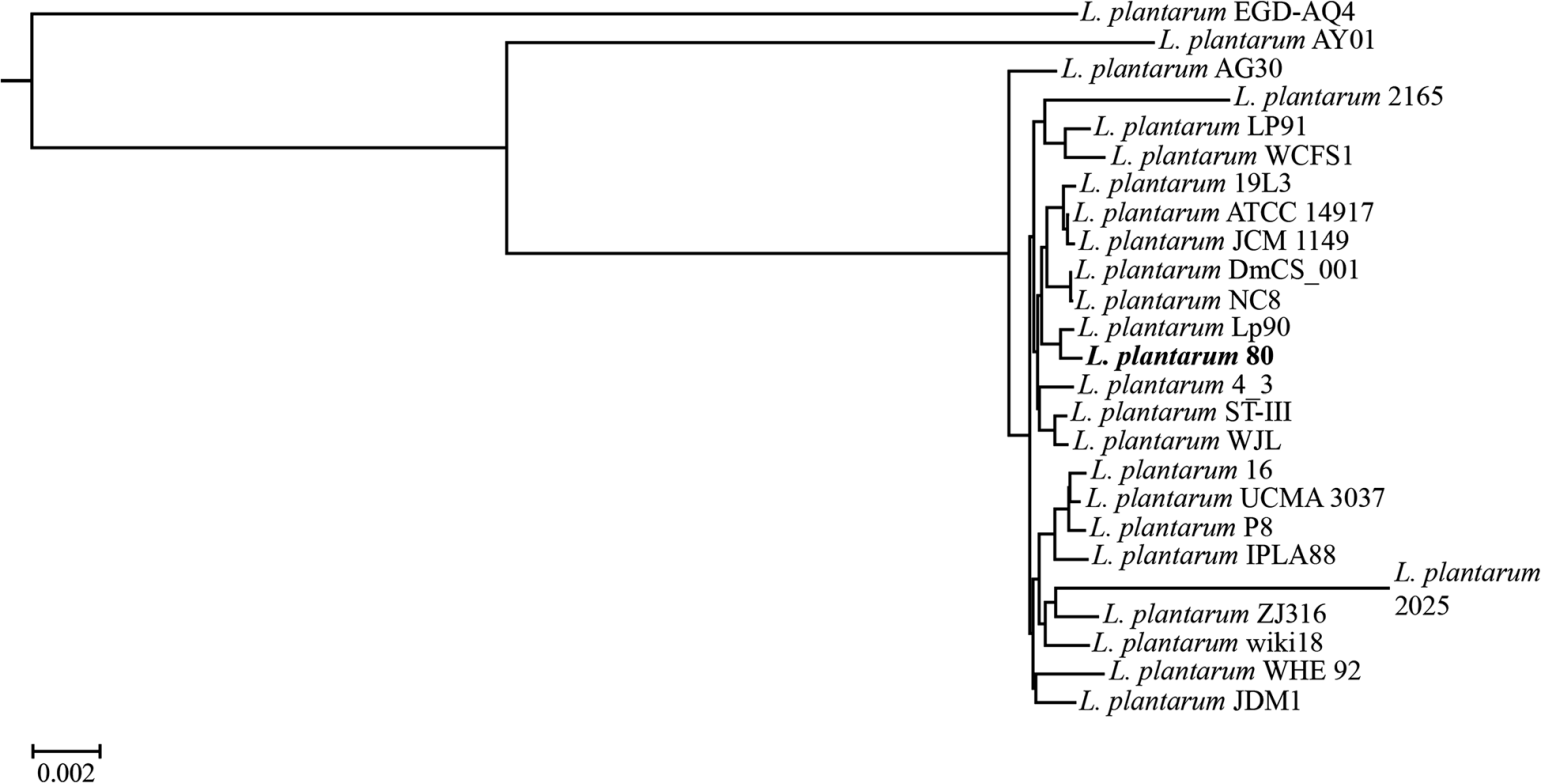
Phylogenetic tree based on all core gene sequences of *Lactobacillus plantarum* available. Multiple sequence alignments of concatenated core gene sequences were calculated using the EDGAR framework, including plasmid sequences. The genome sequence of *L. fermentum* 222 was used as an outgroup

Based on a comparative analysis of 25 *L. plantarum* genome sequences (Additional file 2), the core- and pan-genome of this species encompassed 877 and 8,657 genes, respectively. Construction of a phylogenetic tree based on core gene sequences only (Fig. 5) revealed that two strains (*L. plantarum* EGD-AQ4 and *L. plantarum* AY01) were not part of a larger cluster that contained all other strains. These two strains originated both from fermented food products [38, 45]. Although the phylogenetic distance between the strains was small (Fig. 5), the cocoa bean fermentation isolate *L. plantarum* 80 was grouped together with *L. plantarum* Lp90, an Italian wine isolate [79]. The latter strain possesses the ADI pathway [80] and is tolerant to wine fermentation conditions, such as high sulphite concentrations [81].

## Conclusions

Genomics-based pathway reconstruction of the carbohydrate metabolism and amino acid conversion mechanisms of candidate functional LAB starter culture strains for the cocoa bean fermentation process revealed important insights into their lifestyle, niche adaptations, and metabolic capacities. Although some studies report on comparative genomics of LAB [32, 59, 82], this is the first extensive (comparative) genome sequence analysis of these species, revealing their core- and pan-genome. Identification of the genetic potential of cocoa-derived candidate functional starter cultures obtained from this and other studies [27] in metagenomic data sets will allow further insights into the contribution of functional starter culture strains to cocoa bean fermentation processes [83]. This information is crucial for an improved and rationalised selection of functional starter culture strains for the cocoa bean fermentation process.

## Methods

### Bacterial strains and growth conditions

*Lactobacillus fermentum* 222 and *L. plantarum* 80 were originally isolated from a spontaneous cocoa bean heap fermentation process carried out in Ghana [7]. The strains were stored at −80 °C in de Man-Rogosa-Sharpe (MRS) medium (Oxoid, Basingstoke, United Kingdom), supplemented with 25 % (v/v) glycerol as a cryoprotectant. To obtain cell pellets, the pure strains were grown overnight in MRS medium at 37 °C, followed by cell harvesting through centrifugation (21,036 × *g*, 15 min, 4 °C) of 2-ml cultures.

### DNA extraction and 454 pyrosequencing

Total genomic DNA was extracted from four cell pellets of both *L. fermentum* 222 and *L. plantarum* 80, using a previously described protocol with modifications [84]. A thawed pellet was washed in 1 ml of TES buffer [50 mM Tris base, 1 mM ethylene diamine tetra-acetic acid (EDTA), 6.7 % (m/v) sucrose, pH 8.0] and centrifuged at 2,795 × *g* for 10 min at 4 °C. The resulting pellet was collected and 300 μl of STET buffer [50 mM Tris base, 50 mM EDTA, 8 % (m/v) sucrose, 5 % (m/v) Triton X-100, pH 8.0] was added. The suspension was incubated in the presence of lysozyme (VWR International, Darmstadt, Germany), which was dissolved in TES buffer in a final concentration of 5 U/μl, at 37 °C for 1 h. Thereafter, a chemical treatment with a preheated (37 °C) solution of 20 % (m/v) sodium dodecyl sulphate (SDS; VWR International) in TE buffer (50 mM Tris base, 1 mM EDTA, pH 8.0) and a mechanical treatment with a pinch of glass beads (150–210 μm; Sigma-Aldrich, Steinheim, Germany) was applied, by vortexing the lysate for 60 s and subsequently incubating the mixture at 37 °C for 10 min, followed by a 10-min treatment at 65 °C to inactivate the enzymes. This lysate was extracted with one volume of phenol/chloroform/isoamylalcohol (49.5:49.5:1.0; Sigma-Aldrich). Phases were separated by centrifugation (18,900 × *g*, 5 min) using Phase Lock Gel™ tubes (Eppendorf AG, Hamburg, Germany). DNA was precipitated with 1 ml of isopropanol and 70 μl of NaCl (5 M) on ice and was collected by centrifugation (22,000 × *g*, 30 min, 4 °C). The resulting DNA pellet was washed with 500 μl of a 70 % (v/v) ethanol solution, dried under vacuum, and resuspended in 50 μl of TE buffer. Two μl RNase (10 mg/ml; Sigma-Aldrich) was added, followed by incubation at 37 °C for 10 min. For each strain, the four solutions were pooled and the DNA precipitation and washing steps were repeated. To confirm the identity of the bacterial strains grown, the near full-length 16S rRNA gene was amplified based on the genomic DNA extracted, purified, and sequenced, as described previously [85]. The quality of the genomic DNA was assessed by gel electrophoresis; its quantity was estimated by a fluorescence-based method using the Quant-iT dsDNA assay kit (Invitrogen, Carlsbad, CA, USA) and the DTX800 multimode detector (Beckman Coulter, Pasadena, CA, USA). Next, the isolated genomic DNA was used as template for shotgun pyrosequencing on a genome sequencer (GS) FLX system (Roche Applied Science, Mannheim, Germany) using Titanium chemistry, which was performed by a commercial facility (VIB Nucleomics Core, Leuven, Belgium). A DNA library was constructed for each strain according to the GS FLX rapid library preparation kit (Roche Applied Science). The optimal DNA copy per bead ratio was determined by an emulsion PCR titration, using a GS FLX Titanium SV emPCR kit (Lib-L; Roche Applied Science). A final emulsion PCR for the sequencing runs was performed using the GS FLX Titanium LV emPCR kit (Lib-L; Roche Applied Science). One pyrosequencing run was carried out with the DNA libraries, with each strain occupying half a PicoTiterPlate.

### Genome sequence assembly and annotation

Reads were assembled using Newbler version 2.7 (Roche Applied Science) with default parameters. Automated gene prediction and annotation of the assembled contigs longer than 500 bp was carried out using a local installation of the bacterial genome sequence annotation system GenDB version 2.2, as described previously [27]. Briefly, GLIMMER 2.1 [86] and CRITICA [87] were applied to predict the gene sequences present. Ribosomal binding sites were identified with RBSfinder [88] and tRNA genes were searched for using tRNAscan-SE [89]. The proteins deduced were functionally characterised by REGANOR [90] using automated searches in public databases, including SWISS-PROT [91], Pfam [92], KEGG [93], and TIGRFAM [94]. Additionally, SignalP [95], helix-turn-helix [96], and TMHMM [97] were applied to find signal peptides, helix-turn-helix DNA binding motifs, and transmembrane regions, respectively. Each gene was functionally classified by assigning a cluster of orthologous groups (COG) and a gene ontology (GO) number [98, 99]. To further supplement the annotation, plasmids were identified using the PATRIC database plasmid_seq, virulence factors were searched for by BLASTn (based on nucleotide sequence) analysis using the human pathogenic bacteria virulence factor database (VFDB; [100]), antibiotic resistance genes were searched for by BLASTn analysis using the antibiotic resistance gene annotation database (ARG-ANNOT; [101]), CRISPRs were searched for with CRISPRFinder [102] and CRT [103], prophage sequences were identified by PHAST analysis [104], and BAGEL3 [105] was used to find genes encoding bacteriocins. Based on these annotations, metabolic pathways were reconstructed.

## Data availability

The annotated genome sequences were deposited in the DDBJ/EMBL/GenBank database as sequencing project PRJEB5182 with EMBL accession numbers CBZV010000001-CBZV010000073 for *L. fermentum* 222 and sequencing project PRJEB5195 with EMBL accession numbers CBZW010000001-CBZW010000067 for *L. plantarum* 80.

## Comparative genome sequence analysis

Comparative analysis of the genome sequences of *L. fermentum* 222 and *L. plantarum* 80 with available complete and draft genome sequences of strains of the same species (Additional files 1 and 2) was performed by the EDGAR framework [106] using default parameters. This included identification and classification of orthologous genes, identification of strain-specific genes, and calculation of the pan- and core-genomes. Also within this framework, phylogenetic analyses were performed using PHYLIP [107], thereby relying on an alignment of the core genes of a selection of complete and draft genome sequences of members of the species *L. fermentum* (Additional file 1) and *L. plantarum* (Additional file 2), that was generated using MUSCLE [108] and GBLOCKS [109]. As the publicly available draft genome sequence of *L. fermentum* FTDC 8312 was not annotated, its genome sequence was retrieved from the NCBI data repository, followed by automated annotation using the GenDB platform as described above. For the phylogenetic analysis of the species *L. fermentum*, *L. plantarum* 80 was used as an outgroup; for the phylogenetic analysis of the species *L. plantarum*, *L. fermentum* 222 was used as an outgroup.

## Additional files

Additional file 1:
**Overview of strains of** ***Lactobacillus fermentum*** **that have a complete or draft genome sequence publicly available and that were included in the comparative genome sequence analysis performed using the EDGAR framework.** (DOCX 12 kb) Additional file 2:
**Overview of strains of** ***Lactobacillus plantarum*** **that have a complete or draft genome sequence publicly available and that were included in the comparative genome sequence analysis performed using the EDGAR framework [** 110 **-** 111 **].** (DOCX 14 kb) Additional file 3:
**Enzymes involved in key metabolic pathways of** ***Lactobacillus fermentum*** **222 and** ***L. plantarum*** **80.** (DOCX 20 kb)
