# Supplementary material for: Comparative genome analysis of the candidate functional starter culture strains Lactobacillus fermentum 222 and Lactobacillus plantarum 80 for controlled cocoa bean fermentation processes
Source: BMC Genomics. 2015 Oct 12;16:766. doi: 10.1186/s12864-015-1927-0 (PMC4604094; doi:10.1186/s12864-015-1927-0)
Supplement: Additional file 1: — Overview of strains of Lactobacillus fermentum that have a complete or draft genome sequence publicly available and that were included in the comparative genome sequence analysis performed using the EDGAR framework. (DOCX 12 kb) [file 12864_2015_1927_MOESM1_ESM.docx]

**Overview of strains of *Lactobacillus fermentum* that have a complete or draft genome sequence publicly available and that were included in the comparative genome sequence analysis performed using the EDGAR framework.** In the case that NCBI Genomes is the reference, the authors who submitted the sequence are mentioned.

| **Strain** | **Source of isolation** | **NCBI ID** | **Genome status** | **Reference** |
| --- | --- | --- | --- | --- |
| *L. fermentum* 222 | Ghanaian cocoa bean fermentation | CBZV00000000 | Draft | This study |
| *L. fermentum* 28-3-CHN | Unknown | ACQG00000000 | Draft | Ward *et al.*, 2014, NCBI Genomes |
| *L. fermentum* 3872 | Human milk | AVCT00000000 | Draft | [28] |
| *L. fermentum* ATCC 14931 | Fermented beet | ACGI00000000 | Draft | Qin *et al.*, 2014, NCBI Genomes |
| *L. fermentum* CECT 5716 | Human milk | CP002033 | Complete | [31] |
| *L. fermentum* F-6 | Dairy product | CP005958 | Complete | Gao *et al.*, 2014, NCBI Genomes |
| *L. fermentum* FTDC8312 | Human feces | ASXU00000000 | Draft | Ewe *et al.*, 2014, NCBI Genomes |
| *L. fermentum* IFO 3956 | Fermented plant | AP008937 | Complete | [32] |
| *L. fermentum* Lf1 | Human gut | AWXS00000000 | Draft | [29] |
| *L. fermentum* MTCC 8711 | Yoghurt | AVAB00000000 | Draft | [30] |
| *L. fermentum* NB-22 | Unknown | AYHA00000000 | Draft | Chaplin *et al.*, 2014, NCBI Genomes |
