# Supplementary material for: Comparative genome analysis of the candidate functional starter culture strains Lactobacillus fermentum 222 and Lactobacillus plantarum 80 for controlled cocoa bean fermentation processes
Source: BMC Genomics. 2015 Oct 12;16:766. doi: 10.1186/s12864-015-1927-0 (PMC4604094; doi:10.1186/s12864-015-1927-0)
Supplement: Additional file 2: — Overview of strains of Lactobacillus plantarum that have a complete or draft genome sequence publicly available and that were included in the comparative genome sequence analysis performed using the EDGAR framework [ 110 - 111 ]. (DOCX 14 kb) [file 12864_2015_1927_MOESM2_ESM.docx]

**Overview of strains of *Lactobacillus plantarum* that have a complete or draft genome sequence publicly available and that were included in the comparative genome sequence analysis performed using the EDGAR framework.** In the case that NCBI Genomes is the reference, the authors who submitted the sequence are mentioned.

| **Strain** | **Source of isolation** | **NCBI ID** | **Genome status** | **Reference** |
| --- | --- | --- | --- | --- |
| *L. plantarum* 16 | Malt production steep water | CP006033 | Complete | [40] |
| *L. plantarum* 19L3 | Sheep milk | AWTS00000000 | Draft | [42] |
| *L. plantarum* 2025 | Milk product | AVFJ00000000 | Draft | Karlyshev and Abramov, 2014, NCBI Genomes |
| *L. plantarum* 2165 | Soured milk | AVFI00000000 | Draft | [36] |
| *L. plantarum* 4_3 | Fermented soybean | AYTU00000000 | Draft | Li *et al.*, 2014, NCBI Genomes |
| *L. plantarum* 80 | Ghanaian cocoa bean fermentation | CBZW00000000 | Draft | This study |
| *L. plantarum* AG30 | Sheep rumen | JHWA00000000 | Draft | Kelly *et al.*, 2014, NCBI Genomes |
| *L. plantarum* ATCC 14917 | Pickled cabbage | ACGZ00000000 | Draft | Qin *et al.*, 2014, NCBI Genomes |
| *L. plantarum* AY01 | Goat milk cheese | AVAI00000000 | Draft | [45] |
| *L. plantarum* DmCS_001 | *Drosophila* gut | JOJT00000000 | Draft | [110] |
| *L. plantarum* EGD-AQ4 | Fermented bamboo | AVAQ00000000 | Draft | [38] |
| *L. plantarum* IPLA88 | Sourdough | ASJE00000000 | Draft | [34] |
| *L. plantarum* JCM 1149 | Pickled cabbage | BALV00000000 | Draft | Hattori *et al.*, 2014, NCBI Genomes |
| *L. plantarum* JDM1 | Human gut | CP001617 | Complete | [41] |
| *L. plantarum* Lp90 | Wine | JIBX00000000 | Draft | Lamontanara *et al.*, 2014, NCBI Genomes |
| *L. plantarum* LP91 | Human gut | AXDQ00000000 | Draft | [35] |
| *L. plantarum* P-8 | Unknown | CP005942 | Complete | Gao *et al.*, 2014, NCBI Genomes |
| *L. plantarum* ST-III | Kimchi | CP002222 | Complete | [52] |
| *L. plantarum* UCMA 3037 | Cheese | APHP00000000 | Draft | [46] |
| *L. plantarum* WCFS1 | Human saliva | AL935263 | Complete | [111] |
| *L. plantarum* WHE 92 | Cheese | AWOY00000000 | Draft | [47] |
| *L. plantarum* wikim18 | Kimchi | JMEL00000000 | Draft | [43] |
| *L. plantarum* WJL | *Drosophila* gut | AUTE00000000 | Draft | [44] |
| *L. plantarum* NC8 | Grass silage | AGRI00000000 | Draft | [39] |
| *L. plantarum* ZJ316 | Human feces | CP004082 | Finished | [37] |
