# Supplementary material for: Comparative genome analysis of the candidate functional starter culture strains Lactobacillus fermentum 222 and Lactobacillus plantarum 80 for controlled cocoa bean fermentation processes
Source: BMC Genomics. 2015 Oct 12;16:766. doi: 10.1186/s12864-015-1927-0 (PMC4604094; doi:10.1186/s12864-015-1927-0)
Supplement: Additional file 3: — Enzymes involved in key metabolic pathways of Lactobacillus fermentum 222 and L. plantarum 80. (DOCX 20 kb) [file 12864_2015_1927_MOESM3_ESM.docx]

**Enzymes involved in key metabolic pathways of *Lactobacillus fermentum* 222 and *L. plantarum* 80.**

| **Enzyme (EC number)** | **Gene name** | ***L. fermentum* 222 locus tags** | ***L. plantarum* 80 locus tags** |  |
| --- | --- | --- | --- | --- |
| **Fructose transporters** |  |  |  |  |
| Fructose permease | *frw* | LFER_693 | Not present |  |
| Fructose-specific PEP-dependent PTS | *fru* | LFER_1550-LFER_1552 | LP80_807-LP80_810; LP80_902-LP80_904; LP80_1852-LP80_1854 |  |
| **Homo- and heterolactic fermentation pathways** |  |  |  | |
| Glucokinase (EC 2.7.1.2) | *gck* | LFER_210 | LP80_1299 |  |
| Glucose 6-phosphate dehydrogenase (EC 1.1.1.49) | *g6pd* | LFER_7 | LP80_649 |  |
| 6-Phosphogluconolactonase (EC 3.1.1.31) | *pgl* | LFER_745; LFER_746; LFER_1349 | LP80_1013 |  |
| 6-Phosphogluconate dehydrogenase (EC 1.1.1.44) | *gnd* | LFER_8 | LP80_2615; LP80_1326 |  |
| Ribulose-phosphate epimerase (EC 5.1.3.1) | *rpe* | LFER_166 | LP80_1434 |  |
| Phosphoketolase (EC 4.1.2.9) | *xfp* | LFER_1535 | LP80_668 |  |
| Acetate kinase (EC 2.7.2.1) | *ackA* | LFER_1634 | LP80_342; LP80_1961; LP80_724 |  |
| Phosphate acetyltransferase (EC 2.3.1.8) | *pta* | LFER_1114 | LP80_1740 |  |
| Acetaldehyde dehydrogenase (EC 1.1.1.1) | *adhA* | LFER_492 | LP80_2076 |  |
| Alcohol dehydrogenase (EC 1.1.1.2) | *adhC* | LFER_1797 | LP80_361 |  |
| Glyceraldehyde 3-phosphate dehydrogenase (EC 1.2.1.12) | *gapA* | LFER_1125 | LP80_1723 |  |
| Phosphoglycerate kinase (EC 2.7.2.3) | *pgk* | LFER_1124 | LP80_1724 |  |
| Phosphoglycerate mutase (EC 5.4.2.1) | *pgm* | LFER_900; LFER_1238; LFER_1844; LFER_518; LFER_1608; LFER_1627 | LP80_1795; LP80_3053; LP80_1966; LP80_561; LP80_2511; LP80_967; LP80_211; LP80_269 |  |
| Enolase (EC 4.2.1.11) | *eno* | LFER_1122 | LP80_1726; LP80_2635 |  |
| Pyruvate kinase (EC 2.7.1.40) | *pykA* | LFER_407 | LP80_1280 |  |
| L-lactate dehydrogenase (EC 1.1.1.27) | *ldhA* | LFER_1317; LFER_1048; LFER_1510; LFER_1322 | LP80_527; LP80_101 |  |
| D-lactate dehydrogenase (EC 1.1.1.28) | *ldhB* | LFER_893; LFER_1575; LFER_970; LFER_108 | LP80_2230; LP80_293 |  |
| Glucose 6-phosphate isomerase (EC 5.3.1.9) | *gpi* | Not present | LP80_2834 |  |
| 6-Phosphofructokinase (EC 2.7.1.11) | *pfkA* | Not present | LP80_1281 |  |
| Fructose-bisphosphate aldolase (EC 4.1.2.13) | *fba* | Not present | LP80_362 |  |
| Triose-phosphate isomerase (EC 5.3.1.1) | *tpi* | Not present | LP80_1725 |  |
| **Pyruvate metabolism** |  |  |  |  |
| Pyruvate dehydrogenase E1 (EC 1.2.4.1) | *pdhA* | LFER_1007 - LFER_1008 | LP80_955-LP80_956 |  |
| Pyruvate dehydrogenase E2 (EC 2.3.1.12) | *pdhC* | LFER_1006 | LP80_954 |  |
| Dihydrolipoamide dehydrogenase (EC 1.8.1.4) | *lpdA* | LFER_1005 | LP80_953 |  |
| Pyruvate oxidase (EC 1.2.3.3) | *pox* | LFER_1816 | LP80_2751; LP80_2753; LP80_688; LP80_314 |  |
| Acetyl-CoA hydrolase (EC 3.1.2.1) |  |  |  |  |
| Pyruvate-formate lyase (EC 2.3.1.54) | *pfl* | Not present | LP80_2427 |  |
| α-Acetolactate synthase (EC 2.2.1.6) | *als* | LFER_932 | LP80_185 |  |
| Acetolactate decarboxylase (EC 4.1.1.5) | *aldB* | LFER_649 | LP80_2203 |  |
| Diacetyl reductase (EC 1.1.1.304) | *butA* | LFER_1749; LFER_814 | Not present |  |
| 2,3-Butanediol dehydrogenase (EC 1.1.1.76) | *butC* | LFER_323 | Not present |  |
| **Citrate metabolism** |  |  |  |  |
| Citrate lyase (EC 4.1.3.6) | *citDEF* | LFER_306-LFER_308 | LP80_94-LP80_96 |  |
| Malate dehydrogenase (oxaloacetate-decarboxylating) (EC 1.1.1.38) | *mae* | LFER_303 | LP80_98 |  |
| Fumarase (EC 4.2.1.2) | *fumC* | LFER_627 | LP80_87 |  |
| Fumarate reductase (EC 1.3.5.1) | *frdC* | LFER_626; LFER_1461 | LP80_86 |  |
| Malate dehydrogenase (EC 1.1.1.37) | *mdh* | Not present | LP80_117 |  |
| **Proteolytic system and amino acid conversion pathways** | | |  |  |
| Peptide transporter DtpT | *dtpT* | LFER_978; LFER_1851 | LP80_2471 |  |
| Opp transporter | *oppABCDF* | Not present | LP80_2602-LP80_2606 |  |
| Amino acid transporter | *gln,met, livABCDE* | LFER_1214-LFER_1215; LFER_131-LFER_133; LFER_28-LFER_30; LFER_714-LFER_716; LFER_825-LFER_826; LFER_1300-LFER_1306; LFER_1802-LFER_1803 | LP80_1063-LP80_1067 |  |
| Endopeptidase | *pepF,pepO,pepQ,pepR* | LFER_16; LFER_36 | LP80_693, LP80_1019; LP80_737; LP80_1533; LP80_1887 |  |
| Proline-specific peptidases | *pepI,pepP,pepQ,pepX,pepR* | LFER_875; LFER_186; LFER_1650; LFER_876 | LP80_307; LP80_310; LP80_1408 |  |
| Dipeptidase | *pepD,pepV,pepE* | LFER_993; LFER_1474; LFER_1530; LFER_284 | LP80_222, LP80_366, LP80_1945, LP80_2184; LP80_1298; LP80_2018 |  |
| Tripeptidase | *pepT* | LFER_410 |  |  |
| Aminopeptidase | *pepC,pepN,pepM* | LFER_516; LFER_1895; LFER_1906, LFER_1040 | LP80_242; LP80_1592, LP80_2084 |  |
| Aspartate transaminase | *aspC* | LFER_964, LFER_971 | LP80_2161 |  |
| Aspartase | *aspA* | LFER_136 | LP80_2532 |  |
| Asparaginase | *ans* | Not present | LP80_600 |  |
| Glutamate decarboxylase | *gadB* | Not present | LP80_1511 |  |
| Transaminase | *araT,bcaT* | LFER_109; LFER_138; LFER_336; LFER_1843; LFER_345; LFER_1509 | LP80_857; LP80_294 |  |
| Cystathionine β-lyase | *cbl* | LFER_1309, LFER_1451 | LP80_2922 |  |
| Cysteine kinase | *cysK* | Not present | LP80_2923 |  |
| Serine deaminase | *sda* | LFER_1410, LFER_1411 | LP80_494, LP80_495 |  |
| Serine hydroxymethyltransferase | *shm* | LFER_666 | LP80_845 |  |
| **Arginine deiminase pathway** |  |  |  |  |
| Arginine deiminase | *arcA* | LFER_467 | Not present |  |
| Ornithine transcarbamoyltransferase | *arcB* | LFER_469 | Not present |  |
| Carbamate kinase | *arcC* | LFER_468 | Not present |  |
| Arginine/ornithine antiporter | *arcD* | LFER_466 | Not present |  |
| Transaminase | *arcT* | LFER_464 | Not present |  |
| Ornithine cyclodeaminase (EC 4.3.1.12) | *ocd* | LFER_337 | Not present |  |
| **Alternative electron acceptors** |  |  |  |  |
| NADH-dependent oxidase (EC 1.6.99.3) | *nox* | Not present | LP_1537 |  |
| **Respiration machinery** |  |  |  |  |
| NADH-dependent dehydrogenase (EC 1.6.99.3) |  | Not present | LP80_128 |  |
| *bd*-type cytochrome | *cydABCD* | Not present | LP80_75-LP80_76 |  |
| ATP synthase | *atpABCDEFGH* | LFER_671-LFER_678 | LP80_834-LP80_841 |  |
| Catalase | *katA* | not present | LP80_2743 |  |
|  |  |  |  |  |
